# Attitudes towards homeless people among emergency department teachers and learners: a cross-sectional study of medical students and emergency physicians

**DOI:** 10.1186/1472-6920-13-112

**Published:** 2013-08-23

**Authors:** Alison G Fine, Tony Zhang, Stephen W Hwang

**Affiliations:** 1 Department of Family Medicine, University of Manitoba, Winnipeg, MN, Canada; 2 Department of Internal Medicine, Western University, London, ON, Canada; 3 Centre for Research on Inner City Health, St. Michael’s Hospital, Division of General Internal Medicine, University of Toronto, Toronto, Canada

## Abstract

**Background:**

Medical students’ attitudes and beliefs about homeless people may be shaped by the attitudes of their teachers and one of the most common sites for learning about homeless patients is the emergency department. The objective of this study was to determine if medical students in the preclinical and clinical years and emergency medicine faculty and residents have different attitudes and beliefs about homeless people.

**Methods:**

The Health Professional Attitudes Toward the Homeless Inventory (HPATHI), was administered to all medical students, and emergency medicine physicians and residents at a large academic health sciences center in Canada. The HPATHI examines attitudes, interest and confidence on a 5-point Likert scale. Differences among groups were examined using the Kruskal Wallis test and Pearson’s chi-square test.

**Results:**

The HPATHI was completed by 371 individuals, for an overall response rate of 55%. Analysis of dichotomized median and percentage results revealed 5/18 statements were significant by both methods. On the attitudes subscales physicians and residents as a group were more negative for 2/9 statements and on the confidence subscale more positive for 1/4 statements. The interest subscale achieved overall statistical significance with decreased positive responses among physicians and residents compared to medical students in 2/5 statements.

**Conclusion:**

This study revealed divergences in attitudes, interests and beliefs among medical students and emergency medicine physicians and residents. We offer strategies for training interventions and systemic support of emergency faculty. Emergency medicine physicians can examine their role in the development of medical students through both formal and informal teaching in the emergency department.

## Background

Medicine is a profession that attempts to develop both clinical expertise and ethical behaviour in its students, including an ethic of service to the underserved. Unfortunately, several studies have linked progression through the course of medical education with a rise in cynicism, a decrease in empathy and a decreased interest in caring for the poor [1-4]. Healthcare professionals’ attitudes and beliefs about disadvantaged patients in general and homeless people in particular, can have an important effect on therapeutic interactions. Past studies have found that homeless people’s sense of being unwelcome in health care settings and a lack of appropriate training for medical professionals are major barriers for homeless individuals seeking medical care [5,6].

The evolution of medical students’ attitudes and beliefs towards homeless people is complex and may be shaped by both clinical encounters with homeless patients as well as role-modelling by teachers [4]. The site of these learning experiences with homeless patients is often the emergency department [7]. Homeless populations have very high morbidity, mortality and healthcare needs and often rely on emergency rooms for primary health care rather than using regular sources of health care such as a family doctor or clinic [7-13]. The emergency department is designed to address acute health problems and only limited capabilities are available to deliver preventative care, manage chronic conditions, address socioeconomic factors and arrange appropriate follow-up for homeless patients. Homeless patients who are frequent users of the emergency department can slow acute care and occupy bed space. As a result, medical students often first learn about the care of homeless patients in the emergency department under conditions that may be suboptimal to promote empathetic attitudes towards this population.

The attitudes and beliefs held by teachers and learners during clinical experiences are an important informal component of the medical curriculum. In the emergency department, emergency medicine faculty and residents provide professional role-modelling for medical students in caring for homeless patients. There is minimal research on the attitudes of emergency care providers towards homeless patients. The objective of this study was to determine if medical students in the preclinical and clinical years have different attitudes and beliefs about homeless people compared to emergency medicine faculty and residents.

## Methods

### Setting and study population

This cross-sectional study was conducted in London, Ontario, Canada, a city with a population of approximately 474,786 [14]. Medical students and emergency medicine faculty and residents were recruited at the Schulich School of Medicine and Dentistry, Western University. The London Health Sciences Centre, the hospital network affiliated with the Schulich School of Medicine and Dentistry, encompasses three tertiary care centres with two emergency departments and an urgent care centre. Medical students at this institution undergo two years of preclinical training with minimal patient contact, followed by clinical rotations during the third and fourth years. Emergency medicine residents undergo a five-year training program approved by the Royal College of Physicians and Surgeons of Canada.

Invitations to participate in the survey were emailed to all medical students, emergency medicine faculty and emergency medicine residents. The invitations were followed by email and in-person reminders at one and two weeks. Medical students were asked to complete the survey online or in a paper version. Emergency medicine faculty and residents were asked to complete the survey online. Participation was voluntary and all responses were anonymous.

### Survey instrument

We used the Health Professional Attitudes Toward the Homeless Inventory (HPATHI), an instrument designed to measure health professionals’ attitudes, interest and confidence in delivering healthcare to the homeless [15]. The HPATHI has been previously validated with primary care doctors, primary care residents and third year medical students. The inventory consists of 19 statements, with response options on a 5-point Likert scale (strongly disagree, disagree, neutral, agree, or strongly agree). The statements are grouped into three subscales: attitudes, interest and confidence.

The responses for most items were coded so that scores of 5 (“strongly agree” on a scale of 1 to 5) indicated the most positive attitudes towards homeless people and scores of 1 (“strongly disagree”) indicated the most negative attitudes. However, for certain items agreement indicated more negative attitudes for the inventory. These items are: “Homeless people are lazy”, “Homeless people choose to be homeless”, “I resent the amount of time it takes to see homeless patients” and “I feel overwhelmed by the complexity of the problems that homeless people have”.

For this study, one HPATHI item (“I believe caring for the homeless is not financially viable for my career”) was not included because physicians in Canada work within a publicly-funded universal health insurance system. Under this system, physicians receive the same remuneration for each patient regardless of the patient’s housing status. Thus, we considered it unlikely that physicians would avoid treating homeless patients based on financial considerations.

### Statistical analysis

Median scores and interquartile ranges for each survey item were calculated for medical students in the preclinical years, medical students in the clinical years and emergency medicine faculty and residents. Median scores were calculated for each of the three subscales of attitudes, interest and confidence and the median and interquartile range of the means were reported. The non-parametric Kruskal Wallis test was used to compare responses among the three participant groups. In addition, responses were dichotomized as expressing agreement (“agree” or “strongly agree”) vs. all other responses. The proportions of participants expressing agreement were compared using Pearson’s chi-square tests. All analyses were conducted using SPSS version 17.0 (IBM Corp., Armonk, NY). The study was approved by the London Health Sciences Research Ethics Board.

## Results

Email invitations were sent to 678 individuals and 371 responses were received, for an overall response rate of 55%. The response rates were 54% (178/327) among medical students in the preclinical years, 50% (142/283) among medical students in the clinical years, 76% (19/25) among emergency medicine residents and 74% (32/43) among emergency medicine faculty. The latter two groups were combined for sufficient analytical power and as emergency medicine faculty and residents both mentor medical students in the emergency department.

The median scores and interquartile ranges for each item subscale and the percentages of participants expressing agreement with each item are shown in Table 1 and Table 2. Attitudes towards homeless people were generally positive, with greater than 50% of participants agreeing with statements indicating positive attitudes. In comparisons among the three HPATHI subscales, attitudes tended to be the most positive, followed by the subscale of interest and then confidence.

**Table 1:** Number and percent agreeing or strongly agreeing with statement for HPATHI items by medical training

**HPATHI Item**	**Overall (n = 371)**	**Medical Students (Year 1–2) (n = 178)**	**Medical Students (Year 3–4) (n = 142)**	**ER Residents and Staff (n = 51)**	**p-value**
Attitudes					
Homeless people are victims of circumstances	209 (56.3)	110 (61.8)	79 (55.6)	20 (39.2)	0.016^§^
Homeless people have the right to basic health care	368 (99.2)	176 (98.9)	141 (99.3)	51 (100.0)	0.721
Homelessness is a major problem in our society	311 (83.8)	146 (82.0)	122 (85.9)	43 (84.3)	0.640
I understand that my patients’ priorities may be more important to them than following my medical recommendations^x^	315 (85.1)	155 (87.6)	120 (84.5)	40 (78.4)	0.261
Health-care dollars should be directed toward serving the poor and homeless^x^	253 (68.4)	124 (69.7)	97 (68.8)	32 (62.7)	0.639
Doctors should address the physical and social problems of the homeless	282 (76.0)	144 (80.9)	108 (76.1)	30 (58.8)	0.005^§^
I believe social justice is an important part of health care^†^	289 (78.3)	144 (81.8)	107 (75.4)	38 (74.5)	0.295
Homeless people are lazy	17 (4.58)	9 (5.06)	7 (4.93)	1 (1.96)	0.627
Homeless people choose to be homeless	21 (5.66)	11 (6.18)	6 (4.22)	4 (7.84)	0.579
Interest					
I entered medicine because I want to help those in need^x^	329 (88.9)	158 (88.8)	128 (90.8)	43 (84.3)	0.450
I am interested in working with the underserved^x^	247 (66.8)	122 (68.5)	94 (66.7)	31 (60.8)	0.584
I enjoy addressing psychosocial issues with patients^x^	196 (53.0)	98 (55.4)	83 (58.5)	15 (29.4)	0.001^§^
I enjoy learning about the lives of my homeless patients^x^	147 (39.7)	74 (41.8)	62 (43.7)	11 (21.6)	0.016^§^
I resent the amount of time it takes to see homeless patients^‡^	25 (6.74)	12 (6.74)	11 (7.75)	2 (3.92)	0.646
Confidence					
I am comfortable being a primary care provider for a homeless person with a major mental illness	181 (48.8)	83 (46.6)	77 (54.2)	21 (41.2)	0.202
I feel comfortable being part of a team when providing care to the homeless	323 (87.1)	146 (82.0)	131 (92.3)	46 (90.2)	0.020
I feel comfortable providing care to different minority and cultural groups	337 (90.8)	158 (88.8)	131 (92.3)	48 (94.1)	0.383
I feel overwhelmed by the complexity of the problems that homeless people have	175 (47.17)	90 (50.56)	73 (51.41)	12 (23.53)	0.001^§^

**Table 2:** Median scores and interquartile ranges (IQR) for HPATHI items by medical training

**HPATHI Item**	**Overall (n = 371)**	**Medical students (Year 1–2) (n = 178)**	**Medical students (Year 3–4) (n = 142)**	**ER Residents and staff (n = 51)**	**p-value**
Attitudes	4.0 (3.7-4.2)	4.0 (3.7-4.2)	3.9 (3.7-4.2)	3.9 (3.6-4.1)	0.059
Homeless people are victims of circumstances	4 (3–4)	4 (3–4)	4 (3–4)	3 (3–4)	0.019^§^
Homeless people have the right to basic health care	5 (5–5)	5 (5–5)	5 (4.75-5)	5 (5–5)	0.860
Homelessness is a major problem in our society	4 (4–5)	4 (4–5)	4 (4–4)	4 (4–5)	0.904
I understand that my patients’ priorities may be more important to them than following my medical recommendations^x^	4 (4–4)	4 (4–4)	4 (4–4)	4 (4–4)	0.098
Health-care dollars should be directed toward serving the poor and homeless^x^	4 (3–4)	4 (3–4)	4 (3–4)	4 (3–4)	0.619
Doctors should address the physical and social problems of the homeless	4 (4–4)	4 (4–4)	4 (4–4)	4 (3–4)	0.001^§^
I believe social justice is an important part of health care^†^	4 (4–5)	4 (4–5)	4 (3.75-5)	4 (3–4)	0.192
Homeless people are lazy (reverse)	4 (3–4)	4 (3–4)	4 (3–4)	4 (3–4)	0.641
Homeless people choose to be homeless (reverse)	4 (3–4)	4 (3–4)	4 (3–4)	4 (3–4)	0.813
Interest	3.6 (3.4-4.0)	3.8 (3.4-4.2)	3.7 (3.4-4.0)	3.4 (3.0-3.8)	0.002
I entered medicine because I want to help those in need^x^	4 (4–5)	4 (4–5)	4 (4–5)	4 (4–5)	0.007
I am interested in working with the underserved^x^	4 (3–4)	4 (3–4)	4 (3–4)	4 (3–4)	0.042
I enjoy addressing psychosocial issues with patients^x^	4 (3–4)	4 (3–4)	4 (3–4)	2 (2–4)	<0.001^§^
I enjoy learning about the lives of my homeless patients^x^	3 (3–4)	3 (3–4)	3 (3–4)	3 (2–3)	0.001^§^
I resent the amount of time it takes to see homeless patients (reverse)^‡^	4 (3–4)	3 (3–4)	4 (3–4)	4 (3–4)	0.221
Confidence	3.5 (3.3-4.0)	3.5 (3.3-4.0)	3.5 (3.3-3.8)	3.8 (3.3-4.3)	0.292
I am comfortable being a primary care provider for a homeless person with a major mental illness	3 (2–4)	3 (2–4)	4 (3–4)	3 (2–4)	0.048
I feel comfortable being part of a team when providing care to the homeless	4 (4–5)	4 (4–5)	4 (4–4)	4 (4–5)	0.825
I feel comfortable providing care to different minority and cultural groups	4 (4–5)	4 (4–5)	4 (4–5)	5 (4–5)	0.044
I feel overwhelmed by the complexity of the problems that homeless people have (reverse)	3 (2–3)	2 (2–3)	2 (2–3)	4 (3–4)	<0.001^§^

Scores range from 1 to 5, with 5 indicating the most positive beliefs and attitudes towards homeless people.

Category totals are stated as median scores and interquartile ranges (IQR) for HPATHI items (1 = strongly disagree to 5 = strongly agree).

**Notes**.

^x^Data was missing for 1 respondent.

†Data were missing for 2 respondents.

^‡^ Data were missing for 4 respondents.

§ Data were significant in both median and percentage analysis.

(reverse) indicates a negative statement that has been reverse-coded, so that for every survey item a higher numerical score indicates more positive attitudes towards homeless people.

p < 0.05.

Medical students tended to have more positive attitudes and beliefs about homeless people than emergency medicine faculty and residents. For five HPATHI items, this difference was statistically significant in analyses based on median scores as well as analyses based on percentages expressing agreement. On the attitudes and interests subscales these items were “Homeless people are victims of circumstance,” “Doctors should address the physical and social problems of the homeless,” “I enjoy addressing psychosocial issues with patients,” and “I enjoy learning about the lives of my homeless patients.” For example, with respect to the statement “Homeless people are victims of circumstance,” 62% of pre-clinical medical students expressed agreement or strong agreement, compared to 56% of medical students in the clinical years and only 39% of emergency medicine faculty and residents. On the confidence subscale medical students were significantly more likely to agree with the negative statement “I feel overwhelmed by the complexity of the problems that homeless people have”.

## Discussion

This cross-sectional study on attitudes and beliefs about the homeless provides evidence of multiple areas of divergence between teachers and learners in the emergency department setting. Preclinical medical students were more positive in their attitudes and interests in working with homeless people, which is consistent with the overall positive attitudes and increased empathy early in medical school noted by other studies [2,3,16]. A previous study represented the sole prior information on emergency physicians’ views on the homeless, demonstrating more negative beliefs in emergency physicians compared to psychiatrists [17]. Our study establishes the disparate attitudes between undifferentiated medical students and emergency physicians to enable training interventions. This study has certain implications for medical education. Educational strategies for maintaining empathy among medical students have focused on modelling behaviours, guided and prolonged clinical experiences and curriculum revisions [18-22]. Previous work demonstrated medical students’ observations of physicians’ actions and comments while caring for homeless patients influenced students’ subsequent views [4]. In our study, the negative beliefs that exist towards homeless people among emergency medicine faculty and residents suggest role-modelling may have a detrimental influence on beliefs and behaviours. In contrast, medical students who tend to have more positive attitudes towards homeless patients are more likely to feel overwhelmed by the complexity of the problems of homeless patients. This finding likely reflects medical students’ lesser degree of clinical experience and their unfamiliarity with issues of homelessness. We suggest incorporating health screening and promotion around addictions, mental health and infectious disease to enhance medical students’ understanding. This approach also corresponds to the recommendations of the Public Health and Education Task Force of the Society for Academic Emergency Medicine [23].

Addressing the beliefs of emergency physicians is complex. Negative perceptions may be related to the strain of caring for homeless patients, the influence of medical training, the selective entry of medical students with certain beliefs into the field of emergency medicine, cohort effects or generational differences. High levels of burn-out among emergency physicians may also be contributory [24]. Providing resources to emergency physicians when caring for homeless patients may improve outcomes for patients and reduce emergency physicians’ burden of care. For instance, intensive case management has been shown to reduce emergency department use and result in better health outcomes by connecting homeless clients with available community resources [25-27].

This study has certain limitations. As this is a cross-sectional study, causal inferences should not be made about the longitudinal effects of medical education or clinical experience on respondents’ perceptions. Another limitation is the potential for differences between responders and non-responders due to relatively low response rates among medical students. Social desirability bias could have an important effect on results however we attempted to minimize it through an anonymous survey. Finally, the use of a single study site may limit generalizability to other educational settings.

## Conclusions

Our findings suggest that the perception of unwelcomeness among homeless patients in healthcare encounters may in part reflect negative attitudes towards homeless people in the emergency room. These negative attitudes appear to be more prevalent among faculty than students. Future research could administer the HPATHI to physicians in several specialities to allow comparison of attitudes towards homeless people across multiple settings. In addition, a future study could examine the development of medical students’ attitudes during their training and into the specialty selection process.

Our results demonstrate that in the emergency room, negative attitudes and beliefs about homeless people are more prevalent among teachers than learners. The empathy and interest among medical students may be directed towards providing holistic care and gaining increased insight into the context of homelessness. Among emergency physicians, department case-management for homeless patients may improve care for chronic issues and support physicians in caring for homeless patients.

## Competing interests

The author(s) declare they have no competing interests.

## Authors’ contributions

AF contributed to the conception and design of the study, participated in data collection and analysis and drafted the manuscript. TZ contributed to the conception and design of the study, participated in data collection and analysis and contributed to drafting the manuscript. SH participated in data analysis and revised the manuscript in a substantive manner. All authors have reviewed and approved the final manuscript.

## Authors’ information

SWH is Associate Professor, Division of General Internal Medicine, University of Toronto; Staff Physician, St. Michael’s Hospital, Toronto; and Research Scientist, Centre for Research on Inner City Health, the Keenan Research Centre in the Li Ka Shing Knowledge Institute at St. Michael’s Hospital.

## Pre-publication history

The pre-publication history for this paper can be accessed here:

http://www.biomedcentral.com/1472-6920/13/112/prepub
